# Atypical Antipsychotic Drug Ziprasidone Protects against Rotenone-Induced Neurotoxicity: An In Vitro Study

**DOI:** 10.3390/molecules25184206

**Published:** 2020-09-14

**Authors:** Kazuki Terada, Ayumi Murata, Erina Toki, Shotaro Goto, Hirofumi Yamakawa, Shuichi Setoguchi, Daisuke Watase, Mitsuhisa Koga, Jiro Takata, Kazuhisa Matsunaga, Yoshiharu Karube

**Affiliations:** Faculty of Pharmaceutical Sciences, Fukuoka University, Nanakuma, Jonan-ku, Fukuoka 814-0180, Japan; pp150218@cis.fukuoka-u.ac.jp (A.M.); pd191005@cis.fukuoka-u.ac.jp (E.T.); sgoto@fukuoka-u.ac.jp (S.G.); hyamakawa@adm.fukuoka-u.ac.jp (H.Y.); ssetoguchi@fukuoka-u.ac.jp (S.S.); watase@fukuoka-u.ac.jp (D.W.); kogami@fukuoka-u.ac.jp (M.K.); jtakata@fukuoka-u.ac.jp (J.T.); k-matsu@fukuoka-u.ac.jp (K.M.); karube@fukuoka-u.ac.jp (Y.K.)

**Keywords:** schizophrenia, ziprasidone, rotenone, mitochondrial dysfunction, oxidative stress, neurotoxicity, Nrf2 pathway, antioxidant enzyme

## Abstract

Schizophrenia is a severe, chronic mental illness characterized by delusions, hallucinations, negative symptoms, and cognitive dysfunction. Recently, several studies have demonstrated that the pathogenesis of schizophrenia involves mitochondrial dysfunction and oxidative stress. However, the effect of antipsychotic drugs for these events has been poorly investigated. In the present study, we evaluated the neuroprotective effect of an atypical antipsychotic drug, ziprasidone (ZPD), on rotenone (ROT)-induced neurotoxicity involving oxidative stress in PC12 cells. Our data showed that ZPD treatment promoted the translocation of NF-E2-related factor-2 (Nrf2) from cytoplasm to nucleus and activated the expression of its target genes NAD(P)H quinone oxidoreductase (NQO-1), catalase (CAT), and heme oxygenase (HO-1). Additionally, ZPD prevented ROT-induced cell death and intracellular reactive oxygen species production. Interestingly, the use of serotonin 5-HT_1A_ receptor antagonist 1-(2-methoxyphenyl)-4 (4-(2-phtalimido) butyl) piperazine (NAN-190) completely blocked the protective effect of ZPD against ROT-induced cell death. Our results demonstrate the neuroprotective effect of ZPD against ROT-induced neurotoxicity and suggest that ZPD may be a potential candidate for the prevention of mitochondrial dysfunction and oxidative stress in schizophrenia.

## 1. Introduction

Schizophrenia is a mental disorder characterized by the occurrence of psychotic symptoms, including hallucinations and delusions [1,2,3]. It is prevalent in 1% of the global population and usually emerges in early adulthood [2,4]. To date, many causes of schizophrenia have been elucidated; the most convincing is the dopamine hypothesis [5], which has been the basis for the development and clinical administration of many antipsychotic medications [6]. However, the exact molecular mechanisms underlying the pathogenesis of this disorder remain to be elucidated.

Recent evidence suggests that oxidative stress may play an important role in the pathophysiology of schizophrenia [7,8,9]. Oxidative stress refers to an imbalance of free radicals, such as reactive oxygen species (ROS), which are generated from both normal metabolic processes involving neurotransmitters associated with schizophrenia, such as dopamine and glutamate, and various environmental exposures [10,11,12]. In schizophrenia, dysregulation of free radical metabolism has been suggested, as detected by abnormal activities of critical antioxidant enzymes and other indices of lipid peroxidation in plasma, red blood cells, and cerebrospinal fluid [13]. However, further elucidation of the role of free radicals and antioxidants in schizophrenia and its treatment requires systematic investigation.

In the present study, we used rotenone (ROT), a dopaminergic neurotoxin, to produce oxidative stress. ROT is a cytotoxic compound that specifically inhibits mitochondrial complex I activity and causes free radical generation along with mitochondrial dysfunction [14,15]. Determination of the effects of potential agents on ROT-induced neurotoxicity, both in vitro and in vivo, is a useful approach to delineate their neuroprotective profile [16,17]. In addition, ROT has been reported to induce cell death in cultured rat pheochromocytoma cell line 12 (PC12) and nigral–striatal cocultures [18]. Thus, the ROT toxicity model is relevant for elucidating the pathogenesis of schizophrenia related to not only oxidative stress but also dopaminergic neurons’ dysfunction.

PC12 is a cell line derived from the pheochromocytoma of rat adrenal medulla. It has been widely used as a model system for nerve growth factor (NGF)-induced neuronal-like differentiation [19]. Recently, it has been reported that atypical antipsychotic drugs, namely olanzapine and aripiprazole, reduced oxidative stress through the prevention of ROS generation in PC12 cells [20]. However, the underlying mechanism of these effects is unknown. Likewise, the fundamental mechanisms for the neuroprotective effects leading to the prevention of mitochondrial dysfunction and reduction of oxidative stress have not been elucidated for ziprasidone (ZPD), an atypical antipsychotic drug.

In the present study, we investigated whether the atypical antipsychotic drug ZPD imparts neuroprotective effects on ROT-generated oxidative stress in NGF-induced neuronal PC12 cells. Our findings demonstrate that ZPD protected the neuronal PC12 cells from neurotoxicity by activating the NF-E2-related factor-2 (Nrf2) pathway and this effect was inhibited by serotonin 5-HT_1A_ receptor (5-HT_1A_-R) antagonist.

## 2. Results

### 2.1. Influence of ZPD on Cell Viability

**Figure 1:**
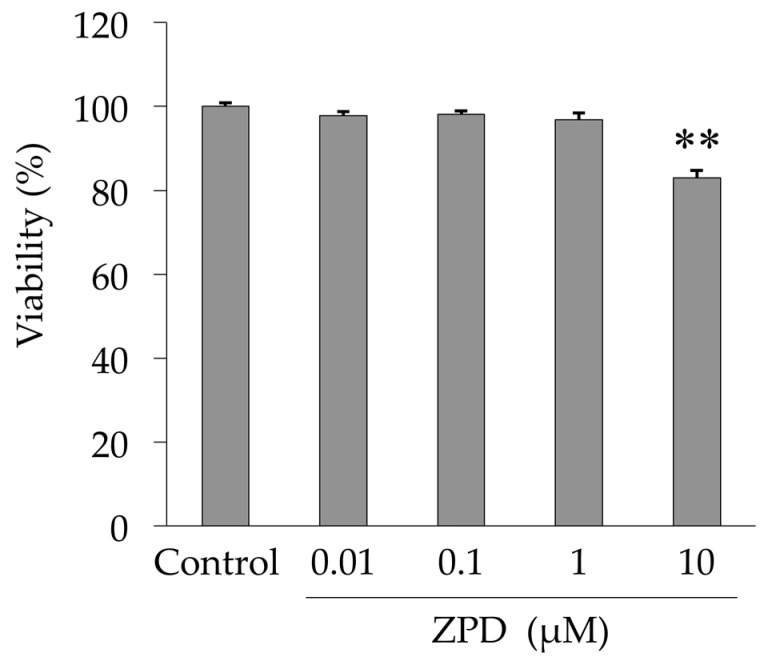
Influence of ziprasidone (ZPD) on viability of PC12 cells. The cells were treated with ZPD for 24 h. Cell viability was determined using CellTiter-Blue assay, and the results are expressed as percentage of the Control value. Experiments were repeated at least three times, and the values represent the mean of three experiments ± standard deviation (SD). ** *p* < 0.01 versus Control.

We investigated the effect of ZPD on the viability of PC12 cells using CellTiter-Blue assay. ZPD did not affect cell proliferation at concentrations below 1 μM, but cytotoxicity was observed at the concentration of 10 μM (Figure 1). Therefore, we used ZPD at the concentrations of 0.01–1 μM for subsequent experiments.

### 2.2. Evaluation of Nrf2 Gene and Protein Expression Modulated by ZPD in PC12 Cells

**Figure 2:**
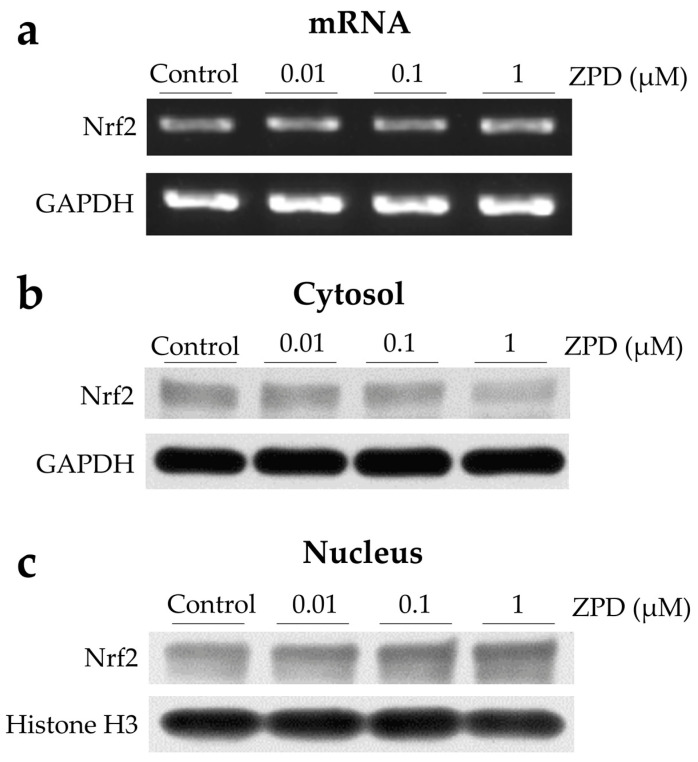
Ziprasidone (ZPD) enhanced nuclear translocation of Nrf2 in differentiated PC12 cells in a dose-dependent manner. Cells were treated with 0.01, 0.1, and 1 µM ZPD for 24 h. Glyceraldehyde 3-phosphate dehydrogenase (GAPDH) and histone H3 were used as internal standards. (**a**) Representative semiquantitative RT-PCR analysis of Nrf2 mRNA expression in differentiated PC12 cells. (**b**,**c**) Western blot analysis of the effect of ZPD on cytoplasmic (b) and nuclear (c) expression of Nrf2 protein. Abbreviations: Nrf2, NF-E2-related factor-2; RT-PCR, reverse transcription polymerase chain reaction.

Nrf2 controls the expression of key components of the glutathione and thioredoxin antioxidant systems, as well as antioxidant enzyme generation, ROS generation, and xenobiotic detoxification [21]. Thus, Nrf2 plays a fundamental role in maintaining the redox homeostasis of the cell. Moreover, it has been reported that Nrf2 translocates to the nucleus, where it activates the transcription of its downstream targets [22]. Hence, to evaluate the effect of ZPD on Nrf2 activation, the mRNA level and protein level of Nrf2 were determined by reverse transcription polymerase chain reaction (RT-PCR) and Western blot analysis, respectively. ZPD treatment did not affect mRNA levels, as indicated by RT-PCR analysis (Figure 2a). In contrast, ZPD decreased the Nrf2 protein expression in a dose-dependent manner in the cytoplasm (Figure 2b) and increased the nuclear Nrf2 protein expression in a dose-dependent manner (Figure 2c), as indicated by Western blot analysis. These results suggest that ZPD enhanced the nuclear translocation of Nrf2, rather than inducing Nrf2 transcription.

### 2.3. ZPD Enhanced the Gene Expression of Antioxidative Enzymes in PC12 Cells

**Figure 3:**
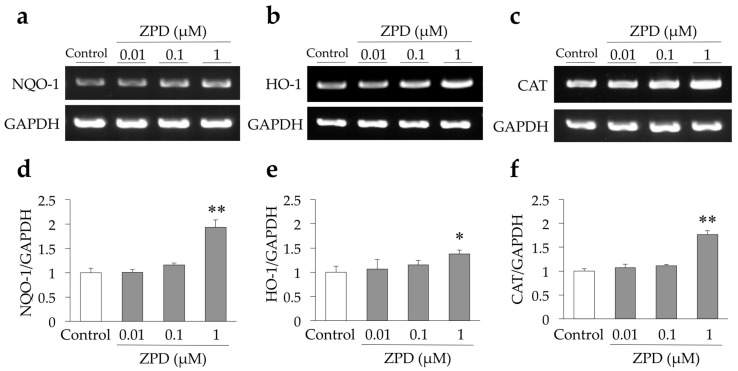
Effect of ziprasidone (ZPD) on the gene expression of NQO-1, HO-1, and CAT in PC12 cells. (**a**–**c**) PC12 cells were treated with 0.01–1 µM ZPD for 24 h. The mRNA expression levels of NQO-1, HO-1, and CAT were determined by semiquantitative RT-PCR as described in Section 4. GAPDH was used as internal control. (**d**–**f**) Quantification of mRNA expression of NQO-1, HO-1, and CAT normalized with GAPDH mRNA intensity (d, NQO-1/GAPDH; e, HO-1/GAPDH; f, CAT/GAPDH). Experiments were repeated at least three times, and the values represent the mean of three experiments ± standard deviation (SD). * *p* < 0.05 compared to Control. ** *p* < 0.01 compared to Control. Abbreviations: CAT, catalase; GAPDH, glyceraldehyde 3-phosphate dehydrogenase; HO-1, heme oxygenase-1; NQO-1, NAD(P)H quinone oxidoreductase; RT-PCR, reverse transcription polymerase chain reaction.

Translocation of Nrf2 from cytoplasm to nucleus is known to induce the expression of several antioxidant enzymes, such as NAD(P)H quinone oxidoreductase (NQO-1), heme oxygenase-1 (HO-1), and catalase (CAT) [23]. NQO-1, HO-1, and CAT play an important role in cellular defense against oxidative stress [24]. Hence, we investigated the expression of these antioxidant enzymes after ZPD treatment. NQO-1 expression was significantly enhanced by treatment with 1 µM ZPD (Figure 3a,d). Similarly, treatment with 1 µM ZPD significantly enhanced the expression of HO-1 (Figure 3b,e) and CAT (Figure 3c,f).

### 2.4. Effect of ZPD on ROT-Induced Neurotoxicity

**Figure 4:**
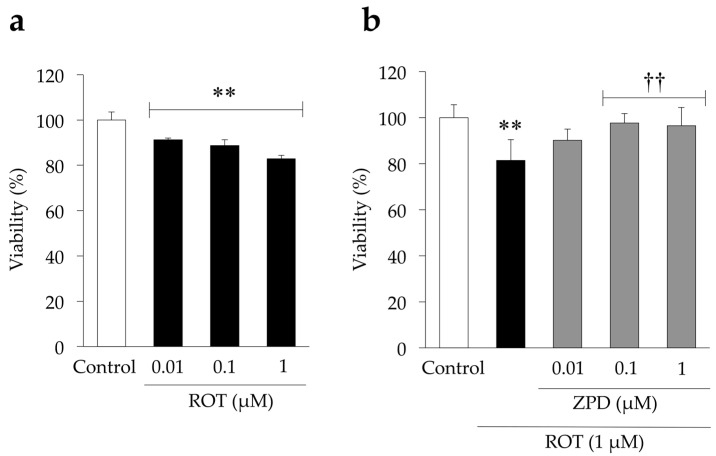
Effects of rotenone (ROT) and ziprasidone (ZPD) on viability of PC12 cells. Cells were incubated with various concentrations of (**a**) ROT and (**b**) ROT and ZPD for 24 h. Cell viability was determined by CellTiter-Blue assay. Experiments were repeated at least three times, and the values represent the mean of three experiments ± standard deviation (SD). ** *p* < 0.01 versus Control. ^††^ *p* < 0.01, compared with ROT-treated cells.

We first determined the neurotoxic effect of ROT by evaluating the survival of PC12 cells. The results revealed that ROT significantly increased cell death in a dose-dependent manner, with a cell survival rate of 82 ± 1.53% being observed in response to 1 µM ROT treatment for 24 h (Figure 4a). Therefore, 1 µM ROT was used in the subsequent experiments. Additionally, we assessed the protective effect of ZPD on ROT-induced cell death. Prior to treatment with ROT to induce cell death, cells were pre-incubated with ZPD for 8 h. As shown in Figure 4b, treatment with 0.1 and 1 µM ZPD increased the viability of PC12 cells significantly.

### 2.5. Effect of ZPD on ROT-Induced Oxidative Stress

**Figure 5:**
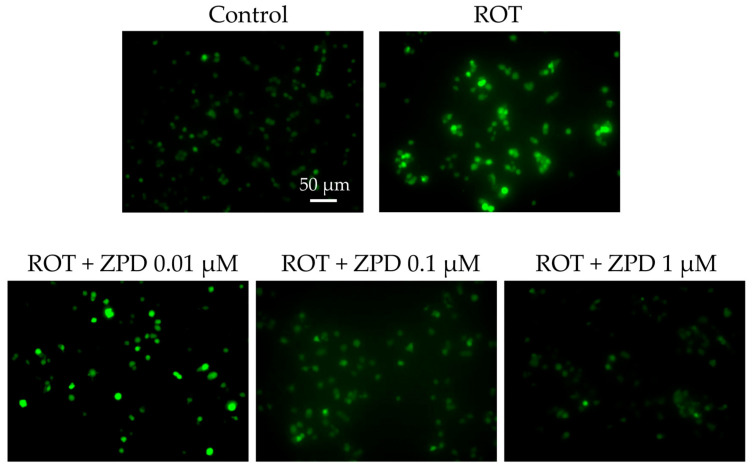
Effect of ziprasidone (ZPD) on rotenone (ROT)-induced reactive oxygen species (ROS) generation. Representative images showing the preventive effect of ZPD against ROT-induced ROS production. Scale bar: 50 μm.

To examine the effect of ZPD on ROT-induced production of intracellular ROS in PC12 cells, ROS production was determined using 2′-7′-dichlorodihydrofluorescein diacetate (DCFH-DA). The generation of intracellular ROS was significantly enhanced by ROT. In contrast, ZPD inhibited ROT-induced ROS production in a dose-dependent manner (Figure 5). These results suggest that ZPD prevented oxidative stress by suppressing the ROT-induced ROS production.

### 2.6. Role of 5-HT_1A_-R in the Protective Effect of ZPD

**Figure 6:**
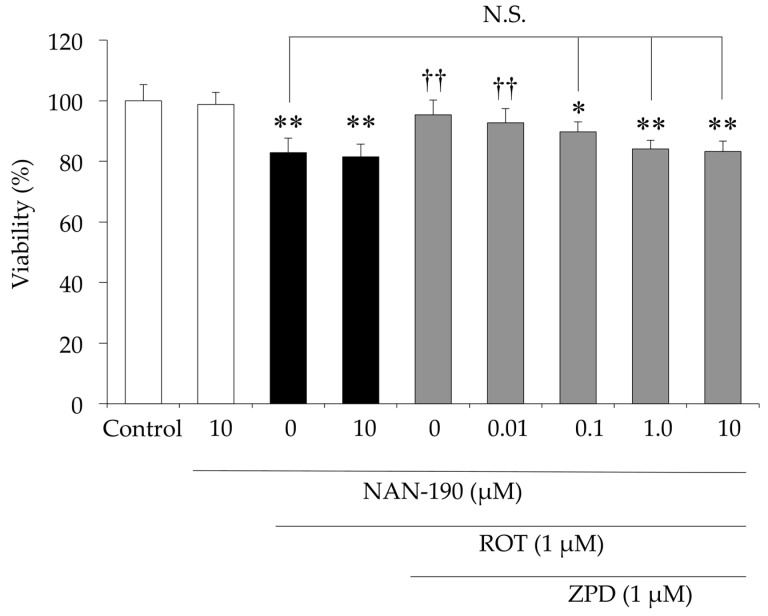
Effect of serotonin 5-HT_1A_ receptor (5-HT_1A_-R) antagonist NAN-190 on the protective effect of ziprasidone (ZPD) against rotenone (ROT)-induced neurotoxicity in PC12 cells. PC12 cells were pre-incubated in the presence or absence of NAN-190 (0–10 μM) for 2 h. ROT (1 µM) and ZPD (1 µM) were added, and the cells were incubated for additional 24 h before CellTiter-Blue assay. Experiments were repeated at least three times, and the values represent the mean of three experiments ± standard deviation (SD). * *p* < 0.05 versus Control. ** *p* < 0.01 versus Control. ^††^ *p* < 0.01, compared to ROT-treated cells. N.S., not significant.

ZPD is a novel antipsychotic with a unique combination of antagonist activities at monoaminergic receptors and transporters and potent agonist activity at 5-HT_1A_-R [25]. Therefore, we investigated the effect of 1-(2-methoxyphenyl)-4(4-(2-phtalimido) butyl) piperazine (NAN-190), a 5-HT_1A_-R antagonist, on the protective effect of ZPD against ROT-induced neurotoxicity. NAN-190 (10 μM) did not inhibit cell death induced by ROT alone (Figure 6). In contrast, the protective effect of ZPD against ROT-induced cell death was inhibited by treatment with NAN-190 in a dose-dependent manner (Figure 6). These findings suggest that prevention of ROT-induced neurotoxicity following treatment with ZPD occurred through 5-HT_1A_-R.

## 3. Discussion

In the present study, we demonstrated that ZPD enhanced the expression of antioxidant enzymes NQO-1, HO-1, and CAT via the Nrf2 pathway. In addition, ZPD prevented ROT-induced neurotoxicity, which was indicated by reduction in cell death and ROS generation in PC12 cells. We also observed that the effect of ZPD was completely blocked by application of a 5-HT_1A_-R antagonist. To the best of our knowledge, this is the first report on neuroprotective effects of ZPD against ROT-induced neurotoxicity with mitochondrial dysfunction and oxidative stress.

In addition to reducing the amount of energy in the brain, mitochondrial dysfunction has been shown to cause neuronal depolarization and alterations in plasticity and circuitry, which can ultimately lead to neuronal death [26,27]. Prabakaran et al. reported that about 50% of the significantly changed proteins in the schizophrenia brain, identified through proteomics study, are associated with mitochondrial function or oxidative stress [28]. Recent clinical studies have shown that antioxidant treatment was effective in ameliorating schizophrenia symptoms. These studies concentrated on compounds such as N-acetylcysteine (NAC), *Ginkgo biloba* extract, and vitamin C [29,30,31]. However, the antioxidant NAC has high water solubility; therefore, it is decomposed easily upon administration, and it cannot pass through the blood–brain barrier. Thus, difficulties exist for the clinical application of NAC [32]. Therefore, identification of viable therapeutic strategies to tackle mitochondrial dysfunction, oxidative stress, and resulting physiological disturbances is required for the treatment of schizophrenia.

Nrf2 has a well-established role as the regulator of key endogenous defenses against oxidative stress in the body [22]. Different types of cellular stresses superimposed on an Nrf2-deficient background have significant detrimental effects on neuronal systems in the brain [33]. Jakel et al. reported that loss of Nrf2-mediated transcription exacerbated vulnerability to neurotoxin 6-hydroxydopamine, an inhibitor of complex I of the mitochondrial respiratory chain, both in cultured neurons and Nrf2-deficient mice in vivo [34]. Thus, there is increasing clinical interest in using Nrf2 activators for therapeutic purposes. Sulforaphane, a potent Nrf2 pathway activator, has been reported to protect the nervous system from many diseases [17], and it is being clinically evaluated for the treatment of schizophrenia and autism disorders [35]. Thus, the ZPD-induced Nrf2 activation may be useful in diseases associated with mitochondrial dysfunction and oxidative stress.

Several antipsychotic drugs have been evaluated for their effect on mitochondrial dysfunction and oxidative stress [20,36]. Typical and atypical antipsychotic drugs have been shown to have different clinical and molecular profiles [37]. Haloperidol is a typical neuroleptic that primarily acts as a dopamine D_2_ receptor antagonist [38]. It has been proposed that ROS play a causative role in neurotoxic effects induced by haloperidol [39]. Similarly, in our study, we observed that haloperidol exacerbated ROT-induced neurotoxicity (data not shown). In contrast, recent studies have suggested that some atypical antipsychotic drugs may have protective effects against oxidative stress. Park et al. reported that atypical antipsychotic drugs ZPD, olanzapine, and aripiprazole exerted antioxidant effects by modulating ROS levels and superoxide dismutase activity [20]. In contrast, Brinholi et al. reported that quetiapine and risperidone lacked antioxidant effects, as indicated by in vitro antioxidant capacity study [40]. Thus, the effects of atypical antipsychotics are not unified, and the underlying mechanisms that affect mitochondria and oxidative stress have not been elucidated. Our results suggest that the neuroprotective effect of ZPD through the effect on mitochondrial dysfunction and oxidative stress is mediated via 5-HT_1A_-R. Several studies employing the agonists of 5-HT_1A_-R have shown their neuroprotective effects in the central nervous system [41,42]. It has been reported that these agents protect against oxidative insults, in part, by upregulating endogenous antioxidant defenses [43]. The atypical antipsychotic drugs, such as aripiprazole and clozapine, have been reported to have partial agonist activity for 5-HT_1A_-R [44,45]. Thus, it may be suggested that these drugs may have the same effect as ZPD. Our study has a few limitations. First, although we have used the PC12 cell line as a model system, this is an in vitro study. Thus, the results cannot be directly extrapolated to in vivo models. To confirm the validity of our results, studies in both in vitro and in vivo model systems should be undertaken in detail. Second, the analysis of gene expression was done using semiquantitative RT-PCR analysis. For more conclusive results, future analyses should be done using quantitative real-time PCR analysis.

## 4. Materials and Methods

### 4.1. Materials

ZPD (ziprasidone hydrochloride) was purchased from Selleck chemicals (Houston, TX, USA). Murine NGF 2.5S (NGF derived from mouse submaxillary glands) was obtained from Alomone Labs (Jerusalem, Israel). ROT was purchased from Sigma-Aldrich (St. Louis, MO, USA). DCFH-DA was obtained from Thermo Fisher Scientific (Waltham, MA, USA). The 5-HT_1A_-R antagonist NAN-190 (NAN-190 hydrobromide) was purchased from Fujifilm (Tokyo, Japan).

### 4.2. Cell Culture

The PC12 cell line was obtained from Riken Cell Bank (Ibaraki, Japan). The cells were maintained in Dulbecco’s modified Eagle’s medium/F-12 supplemented with 10% (*v*/*v*) fetal bovine serum (FBS; Gibco, Life Technologies, Franklin Lakes, NJ, USA) and 1% (*v*/*v*) penicillin–streptomycin. Cells were kept in an incubator at 37 °C in an atmosphere of 5% CO_2_/95% air. For the ROT treatment, cells were seeded in cell culture multi-well plates (Thermo Scientific, Nunc, Naerum, Denmark) for 24 h and then treated with fresh medium containing NGF (final concentration of 50 ng/mL) for 48 h. Then, the cells were treated with ROT for 24 h to induce neurotoxicity. Cells were pretreated with ZPD for 8 h prior to exposure to ROT in the presence of NGF. The control group was treated with the same medium without ROT and ZPD.

### 4.3. Cell Viability Assay

PC12 cells were seeded into 96-well plates (Thermo Scientific, Nunc) at a density of 1.0 × 10^4^ cells/well for 24 h. Following treatment, cell viability was assessed using the cell proliferation reagent CellTiter-Blue (Promega, Southampton, UK) according to the manufacturer’s instructions. Briefly, the culture medium was removed from the wells after treatment, and 100 µL of medium containing 10 µL CellTiter-Blue was added to each well. Fluorescence intensity (excitation 560 nm, emission 590 nm) was determined after 4 h.

### 4.4. Semiquantitative RT-PCR Analysis

RT-PCR was used to analyze the levels of Nrf2, NQO-1, HO-1, CAT, and GAPDH mRNA. GAPDH was used as an internal standard. Total RNA was isolated from 5 × 10^6^ PC12 cells in the logarithmic phase using RNAiso Plus (Takara, Shiga, Japan) according to the manufacturer’s instructions. Reverse transcription was done using ReverTra Ace Master Mix from Toyobo (Osaka, Japan) following the manufacturer’s instructions. Semiquantitative RT-PCR was performed with a thermal cycler system (Bio-Rad) using PrimeSTAR GXL DNA polymerase (Takara, Shiga, Japan). The primers used for PCR are listed in Table 1. The RT-PCR products were separated on 2% agarose gel, and the intensity of each band was quantified using SynGene software (SynGene, Cambridge, UK) and expressed in arbitrary units (GeneGenius Super 12, Syngene, Cambridge, UK).

### 4.5. Western Blot Analysis

Western blot analyses were performed as previously described [19]. The subcellular fractions (cytosolic and nuclear fractions) were separated using a nuclear/cytosolic fractionation kit (Cell Biolabs, San Diego, CA, USA) according to the manufacturer’s instructions. Protein samples containing 20 μg of total protein were separated via electrophoresis with 4–15% sodium dodecyl sulfate (SDS)–polyacrylamide gels, after which they were transferred onto polyvinylidene fluoride (PVDF) membrane (Bio-Rad, Hercules, CA, USA). After transfer, PVDF membrane was blocked with 5% bovine serum albumin (Fujifilm) in TBS containing 0.1% Tween-20 at room temperature for 1 h. Immunoblotting was then performed using primary antibodies against Nrf2 (1:1000, Proteintech, Rosment, IL, USA), GAPDH (1:5000, Cell Signaling Technology, Danvers, MA, USA), and histone H3 (1:2000, Cell Signaling Technology). GAPDH and histone H3 were used as internal standards. Horseradish peroxidase conjugated secondary antibody was used to detect immunoreactivity (Amersham Pharmacia Biotech, Piscataway, NJ, USA), which was visualized using enhanced chemiluminescence Western blotting detection reagents (Amersham Pharmacia Biotech) and RX-U Fuji X-ray film (Fujifilm).

### 4.6. Detection of ROS Production

The levels of intracellular hydrogen peroxide and other peroxides in PC12 cells were estimated by loading cells with DCFH-DA as described previously [46]. In brief, the PC12 cells were washed with PBS (pH 7.4) and then loaded with 10 µM DCFH-DA for 30 min at 37 °C. The images were captured using a fluorescence microscope (BZ-X810, Keyence Co, Osaka, Japan).

### 4.7. Statistical Analysis

Quantitative data are presented as mean ± standard deviation (SD). Statistical analyses of quantitative data were performed using analysis of variance (ANOVA) followed by Tukey’s post hoc test. The differences with *p* < 0.05 were considered statistically significant.

## 5. Conclusions

The present study demonstrated that ZPD protected against ROT-induced neurotoxicity. Our findings suggest that the underlying molecular mechanism for this effect of ZPD was enhanced translocation of Nrf2 from cytoplasm to nucleus after the triggering of signaling cascade through 5-HT_1A_-R. Since oxidative stress and mitochondrial dysfunction have been associated with the pathophysiology of schizophrenia, the increased expression of antioxidant enzymes elicited by ZPD observed in this study may be an effective and potential therapeutic strategy for the treatment of schizophrenia.

## Figures and Tables

**Table 1:** Primer sequences for RT-PCR analyses.

Gene	Accession No.	Sense (5′-3′)	Antisense (5′-3′)
Nrf2	NM_031789	GGACCTAAAGCACAGCCAAC	ATCTCTGGTCTGCTGCAGAG
NQO-1	NM_017000	ATGGGAGGTGGTCGAATCTG	TCTCCAGACGCTTCTTCCAC
HO-1	NM_012580	CTTACACACCAGCCACACAG	ACTGAGTGTGAGGACCCATC
CAT	NM_012520	GATGAAGCAGTGGAAGGAGC	TCGGTCGCTGAACAAGAAAG
GAPDH	NM_017008	AGGCTGAGAATGGGAAGCTG	TAGGAACACGGAAGGCCATG

Abbreviations: CAT, catalase; GAPDH, glyceraldehyde 3-phosphate dehydrogenase; HO-1, heme oxygenase-1; NQO-1, NAD(P)H quinone oxidoreductase; Nrf2, NF-E2-related factor-2; RT-PCR, reverse transcription polymerase chain reaction.

## Abbreviations

ROS, reactive oxygen species; ROT, rotenone; PC12, rat pheochromocytoma cell line 12; NGF, nerve growth factor; ZPD, ziprasidone; Nrf2, NF-E2-related factor-2; 5-HT_1A_-R, serotonin 5-HT_1A_ receptor; RT-PCR, reverse transcription polymerase chain reaction; GAPDH, glyceraldehyde 3-phosphate dehydrogenase; NQO-1, NAD(P)H quinone oxidoreductase; HO-1, heme oxygenase-1; CAT, catalase; DCFH-DA, 2′-7′-dichlorodihydrofluorescein diacetate.

## References

[1] Goadsby P.J., Burke D. (1994). Deficits in the function of small and large afferent fibers in confirmed cases of carpal tunnel syndrome. Muscle Nerve.

[2] Perälä J., Suvisaari J., Saarni S.I., Kuoppasalmi K., Isometsä E.T., Pirkola S., Partonen T., Tuulio-Henriksson A., Hintikka J., Kieseppä T. (2007). Lifetime Prevalence of Psychotic and Bipolar I Disorders in a General Population. Arch. Gen. Psychiatry.

[3] Tamminga C.A., Holcomb H.H. (2004). Phenotype of schizophrenia: A review and formulation. Mol. Psychiatry.

[4] Johnstone E.C., Jablensky A., Sartorius N., Ernberg G., Anker M., Korten A., Cooper J.E., Day R., Bertelsen A. (1991). Schizophrenia: Manifestations, Incidence and Course in Different Cultures. WHO Ten-Country Study.

[5] Carlsson A., Lindqvist M. (2009). Effect of Chlorpromazine or Haloperidol on Formation of 3-Methoxytyramine and Normetanephrine in Mouse Brain. Acta Pharmacol. Toxicol. (Copenh).

[6] Seeman P., Kapur S. (2000). Schizophrenia: More dopamine, more D2 receptors. Proc. Natl. Acad. Sci. USA.

[7] Zhang M., Zhao Z., He L., Wan C. (2010). A meta-analysis of oxidative stress markers in schizophrenia. Sci. China Life Sci..

[8] Flatow J., Buckley P., Miller B.J. (2013). Meta-analysis of oxidative stress in schizophrenia. Boil. Psychiatry.

[9] Brennand K.J., Landek-Salgado M.A., Sawa A. (2014). Modeling Heterogeneous Patients with a Clinical Diagnosis of Schizophrenia With Induced Pluripotent Stem Cells. Boil. Psychiatry.

[10] Hirose K., Chan P.H. (1993). Blockade of glutamate excitotoxicity and its clinical applications. Neurochem. Res..

[11] Kohen R., Nyska A. (2002). Invited Review: Oxidation of Biological Systems: Oxidative Stress Phenomena, Antioxidants, Redox Reactions, and Methods for Their Quantification. Toxicol. Pathol..

[12] Grima G. (2003). Dopamine-induced oxidative stress in neurons with glutathione deficit: Implication for schizophrenia. Schizophr. Res..

[13] Reddy R., Yao J.K. (1997). Free radical pathology in schizophrenia: A review. Schizophr. Res..

[14] Li N., Ragheb K., Lawler G., Sturgis J., Rajwa B., Melendez J.A., Robinson J.P. (2002). Mitochondrial Complex I Inhibitor Rotenone Induces Apoptosis through Enhancing Mitochondrial Reactive Oxygen Species Production. J. Boil. Chem..

[15] Won J.-H., Park S., Hong S., Son S., Yu J.-W. (2015). Rotenone-induced Impairment of Mitochondrial Electron Transport Chain Confers a Selective Priming Signal for NLRP3 Inflammasome Activation. J. Boil. Chem..

[16] Ren Y., Feng J. (2007). Rotenone selectively kills serotonergic neurons through a microtubule-dependent mechanism. J. Neurochem..

[17] Zhou Q., Chen B., Wang X., Wu L., Yang Y., Cheng X., Hu Z., Cai X., Yang J., Sun X. (2016). Sulforaphane protects against rotenone-induced neurotoxicity in vivo: Involvement of the mTOR, Nrf2 and autophagy pathways. Sci. Rep..

[18] Siebert A., Desai V., Chandrasekaran K., Fiskum G., Jafri M.S. (2009). Nrf2 activators provide neuroprotection against 6-hydroxydopamine toxicity in rat organotypic nigrostriatal cocultures. J. Neurosci. Res..

[19] Terada K., Migita K., Matsushima Y., Sugimoto Y., Kamei C., Matsumoto T., Mori M., Matsunaga K., Takata J., Karube Y. (2018). Cholinesterase inhibitor rivastigmine enhances nerve growth factor-induced neurite outgrowth in PC12 cells via sigma-1 and sigma-2 receptors. PLoS ONE.

[20] Park S.-W., Lee C.H., Lee J.G., Kim L.W., Shin B.S., Lee B.J., Kim Y.H. (2011). Protective effects of atypical antipsychotic drugs against MPP+-induced oxidative stress in PC12 cells. Neurosci. Res..

[21] Yama K., Sato K., Abe N., Murao Y., Tatsunami R., Tampo Y. (2014). Epalrestat increases glutathione, thioredoxin, and heme oxygenase-1 by stimulating Nrf2 pathway in endothelial cells. Redox Boil..

[22] Tonelli C., Chio I.I.C., Tuveson D.A. (2018). Transcriptional Regulation by Nrf2. Antioxid. Redox Signal..

[23] Zhao Y., Song W., Wang Z., Wang Z., Jin X., Xu J., Bai L., Li Y., Cui J., Cai L. (2018). Resveratrol attenuates testicular apoptosis in type 1 diabetic mice: Role of Akt-mediated Nrf2 activation and p62-dependent Keap1 degradation. Redox Boil..

[24] De Vries H.E., Witte M., Hondius D., Rozemuller A.J., Drukarch B., Hoozemans J.J.M., Van Horssen J. (2008). Nrf2-induced antioxidant protection: A promising target to counteract ROS-mediated damage in neurodegenerative disease?. Free. Radic. Boil. Med..

[25] Schmidt A.W., Lebel L.A., Howard H.R., Zorn S.H. (2001). Ziprasidone: A novel antipsychotic agent with a unique human receptor binding profile. Eur. J. Pharmacol..

[26] Maurer I., Zierz S., Möller H.-J. (2001). Evidence for a mitochondrial oxidative phosphorylation defect in brains from patients with schizophrenia. Schizophr. Res..

[27] Zhao X.-Y., Lu M.-H., Yuan D.-J., Xu D.-E., Yao P.-P., Ji W.-L., Chen H., Liu W.-L., Yan C.-X., Xia Y.-Y. (2019). Mitochondrial Dysfunction in Neural Injury. Front. Mol. Neurosci..

[28] Prabakaran S., Swatton J.E., Ryan M.M., Huffaker S.J., Huang J.-J., Griffin J.L., Wayland M., Freeman T., Dudbridge F., Lilley K.S. (2004). Mitochondrial dysfunction in schizophrenia: Evidence for compromised brain metabolism and oxidative stress. Mol. Psychiatry.

[29] Atmaca M., Tezcan E., Kuloglu M., Ustundag B., Kirtas O. (2005). The effect of extract of ginkgo biloba addition to olanzapine on therapeutic effect and antioxidant enzyme levels in patients with schizophrenia. Psychiatry Clin. Neurosci..

[30] Dakhale G.N., Khanzode S.D., Saoji A., Khanzode S.S. (2005). Supplementation of vitamin C with atypical antipsychotics reduces oxidative stress and improves the outcome of schizophrenia. Psychopharmacology.

[31] Berk M., Copolov D., Dean O., Lu K., Jeavons S., Schapkaitz I., Anderson-Hunt M., Judd F., Katz F., Katz P. (2008). N-Acetyl Cysteine as a Glutathione Precursor for Schizophrenia—A Double-Blind, Randomized, Placebo-Controlled Trial. Boil. Psychiatry.

[32] Sunitha K., Hemshekhar M., Thushara R.M., Santhosh M.S., Yariswamy M., Kemparaju K., Girish K.S. (2013). N-Acetylcysteine amide: A derivative to fulfill the promises of N-Acetylcysteine. Free. Radic. Res..

[33] Ramsey C.P., Glass C.A., Montgomery M.B., Lindl K.A., Ritson G.P., Chia L.A., Hamilton R.L., Chu C.T., Jordan-Sciutto K.L. (2007). Expression of Nrf2 in Neurodegenerative Diseases. J. Neuropathol. Exp. Neurol..

[34] Jakel R.J., Townsend J.A., Kraft A.D., Johnson J.A. (2007). Nrf2-mediated protection against 6-hydroxydopamine. Brain Res..

[35] Shiina A., Kanahara N., Sasaki T., Oda Y., Hashimoto T., Hasegawa T., Yoshida T., Iyo M., Hashimoto K. (2015). An Open Study of Sulforaphane-rich Broccoli Sprout Extract in Patients with Schizophrenia. Clin. Psychopharmacol. Neurosci..

[36] Dietrich-Muszalska A., Kolińska-Łukaszuk J. (2018). Comparative effects of aripiprazole and selected antipsychotic drugs on lipid peroxidation in plasma. Psychiatry Clin. Neurosci..

[37] Assis L.C., Scaini G., Castro A.A., Comim C.M., Streck E.L., Quevedo J., Di-Pietro P.B. (2007). Effect of Antipsychotics on Creatine Kinase Activity in Rat Brain. Basic Clin. Pharmacol. Toxicol..

[38] Gardner D.M., Baldessarini R.J., Waraich P. (2005). Modern antipsychotic drugs: A critical overview. Can. Med. Assoc. J..

[39] Polydoro M., Schröder N., Lima M.N.M., Caldana F., Laranja D.C., Bromberg E., Roesler R., Quevedo J., Moreira J.C.F., Dal-Pizzol F. (2004). Haloperidol- and clozapine-induced oxidative stress in the rat brain. Pharmacol. Biochem. Behav..

[40] Brinholi F.F., De Farias C.C., Bonifacio K.L., Higachi L., Casagrande R., Moreira E.G., Barbosa D.S. (2016). Clozapine and olanzapine are better antioxidants than haloperidol, quetiapine, risperidone and ziprasidone in in vitro models. Biomed. Pharmacother..

[41] Mauler F., Horváth E. (2005). Neuroprotective Efficacy of Repinotan HCl, a 5-HT1A Receptor Agonist, in Animal Models of Stroke and Traumatic Brain Injury. Br. J. Pharmacol..

[42] Salazar-Colocho P., Del Río J., Frechilla D. (2008). Neuroprotective effects of serotonin 5-HT1A receptor activation against ischemic cell damage in gerbil hippocampus: Involvement of NMDA receptor NR1 subunit and BDNF. Brain Res..

[43] Collier R.J., Patel Y., Martin E.A., Dembinska O., Hellberg M., Krueger D.S., Kapin M.A., Romano C. (2011). Agonists at the Serotonin Receptor (5-HT1A) Protect the Retina from Severe Photo-Oxidative Stress. Investig. Opthalmol. Vis. Sci..

[44] Dekeyne A., Rivet J.-M., Gobert A., Millan M.J. (2001). Generalization of serotonin (5-HT)1A agonists and the antipsychotics, clozapine, ziprasidone and S16924, but not haloperidol, to the discriminative stimuli elicited by PD128,907 and 7-OH-DPAT. Neuropharmacology.

[45] Jordan S., Koprivica V., Chen R., Tottori K., Kikuchi T., Altar C.A. (2002). The antipsychotic aripiprazole is a potent, partial agonist at the human 5-HT1A receptor. Eur. J. Pharmacol..

[46] Tong Y., Bai L., Gong R., Chuan J., Duan X., Zhu Y. (2018). Shikonin Protects PC12 Cells Against β-amyloid Peptide-Induced Cell Injury Through Antioxidant and Antiapoptotic Activities. Sci. Rep..

